# Climate change and bird extinctions in the Amazon

**DOI:** 10.1371/journal.pone.0236103

**Published:** 2020-07-17

**Authors:** Kauê Felippe de Moraes, Marcos Pérsio Dantas Santos, Gabriela Silva Ribeiro Gonçalves, Geovana Linhares de Oliveira, Leticia Braga Gomes, Marcela Guimarães Moreira Lima

**Affiliations:** 1 Programa de Pós-Graduação em Ecologia, Universidade Federal do Pará, Belém, Brasil; 2 Laboratório de Biogeografia da Conservação e Macroecologia, Universidade Federal do Pará, Belém, Brasil; 3 Programa de Pós-Graduação em Zoologia, Universidade Federal do Pará, Belém, Brasil; Instituto Federal de Educacao Ciencia e Tecnologia Goiano - Campus Urutai, BRAZIL

## Abstract

In recent years, carbon dioxide emissions have been potentiated by several anthropogenic processes that culminate in climate change, which in turn directly threatens biodiversity and the resilience of natural ecosystems. Tropical rainforests are among the most impacted biological realms. The Belém endemism center, which is one of the several endemism centers in Amazon, is located in the most affected area within the so-called “Deforestation Arc.” Moreover, this region harbors a high concentration of Amazonian endangered bird species, of which 56% of them are considered to be under the threat of extinction. In this work, we sought to evaluate the current and future impacts of both climate change and deforestation on the distribution of endemic birds in the Belém Area of Endemism (BEA). Thus, we generated species distribution models for the 16 endemic bird species considering the current and two future gas emission scenarios (optimistic and pessimistic). We also evaluated climate change impacts on these birds in three different dispersal contexts. Our results indicate that BAE, the endemic taxa will lose an average of 73% of suitable areas by 2050. At least six of these birds species will have less than 10% or no future suitable habitat in all emission scenarios. One of the main mechanisms used to mitigate the impacts of climate change on these species in the near future is to assess the current system of protected areas. It is necessary to ensure that these areas will continue being effective in conserving these species even under climate change. The “Gurupi Mosaic” and the “Rio-Capim” watershed are areas of great importance because they are considered climate refuges according to our study. Thus, conservation efforts should be directed to the maintenance and preservation of these two large remnants of vegetation in addition to creating ecological corridors between them.

## Introduction

In recent years, carbon dioxide emissions have been potentiated by various anthropogenic processes, leading to an increase in the Earth’s average temperature [1,2,3,4]. Assessment reports from the *Intergovernmental Panel on Climate Change* (IPCC) have indicated an alarming future as a consequence of these processes. As predicted, climate change brings with it transformations in global climate patterns, such as the melting of both glaciers and permafrost, as well as changes in precipitation regimes [5,6]. Altogether, these transformations threaten biodiversity and the resilience of natural ecosystems [7] by, for instance, decreasing the number of suitable habitats, altering ecological interactions among species, thus, negatively impacting the ecosystem services [8,9,10,11,12,13].

The relationship between climate change and deforestation can aggravate their negative impacts on natural ecosystems [14]. The increase in global average temperatures and longer drought periods, together with habitat reduction and fragmentation, can expose species to an even more vulnerable situation [15]. Some climate change projections predict considerable impacts on species distribution and, consequently, loss of ecosystem functions [16,17]. Moreover, the areas affected by the effects of climate change are under significant threat from anthropogenic activities that aggravate deforestation [18,19].

Given this climate change scenario and its implications for biodiversity, tropical rainforests are among the most impacted areas [20]. Several studies claim a possible “savannization” in eastern Amazon due to more prolonged drought periods in this region [21,22]. Such lengthier drought periods have directly affected the diversity of the flora [4,23] and influenced the distributions of forest-dependent species [24]. Also, the Amazon forest has been affected by various anthropogenic activities, such as logging, large-scale mechanized agriculture, mining, larger livestock populations, and infrastructure expansion [25,26,27,28,29]. Although these activities affect several Amazon regions, the results of these impacts vary between its areas of endemism [30]. Areas of endemism delimited by large rivers are an essential biogeographic pattern in the Amazon. They form a mosaic with a complex evolutionary history [27,30], containing biotas and unique ecological processes, which are the result of several speciation events [31]. Five of the eight endemism centers from the Amazon proposed by Silva et al. [30] are located in the Amazonian region known as the “Deforestation Arc,” where 75% of all deforestation areas are concentrated [28,32,33]. Among these, the Belém Area of Endemism (BAE) is the most impacted, with approximately 76% of its total area already deforested [27].

Currently, BAE has a large concentration of endangered Amazonian bird species, of which 56% of the region’s endemic bird species are endangered [34,35]. Birds are considered key organisms for maintaining the ecological balance within a habitat mainly because of their seed dispersal capabilities [36] and control of insect populations [37]. They also serve as efficient bioindicators due to their sensitivity to environmental change [38]. Thus, they are excellent models for understanding the effects of future climate change and for outlining both conservation and maintenance strategies of biodiversity and ecosystem services.

Species distribution models (SDMs) are commonly used tools that are efficacious in the assessment of the effects of climate change on biodiversity [39,40,41,42,43,44]. These models are based on Grinnell’s [45] niche concept, which describes the ecological niche as the distribution unit occupied by a species, where individuals are constrained by both physical and climatic variables. Thus, based on species occurrence data, and local climatic conditions, it is possible to define the species’ climatic tolerance and predict regions of potential climatic suitability to them [46]. SDMs are also used to identify climate refuge areas, which are habitats that maintain their climate patterns over time amid areas where the climate is altered [47]. In these areas, the species tend to have conserved niches, as they are not expected to adapt quickly to new climatic conditions [48,49]. Therefore, to identify these climate refuges is of paramount importance, as these areas have the necessary resources for the long-term maintenance of the species populations, even in the face of the foreseen climate changes [47,50,51,52,53]. Such assessments are fundamental, especially in landscapes undergoing fast modification [54] and conservation opportunities become limited, and natural lands are converted into agriculture and urban areas, as is the case of the BAE.

Hence, we aimed to answer the following question: What are the current and future impacts of climate change and deforestation on the distribution of endemic birds in the Belém Area of Endemism?

## Material and methods

### Study area

**Fig 1:**
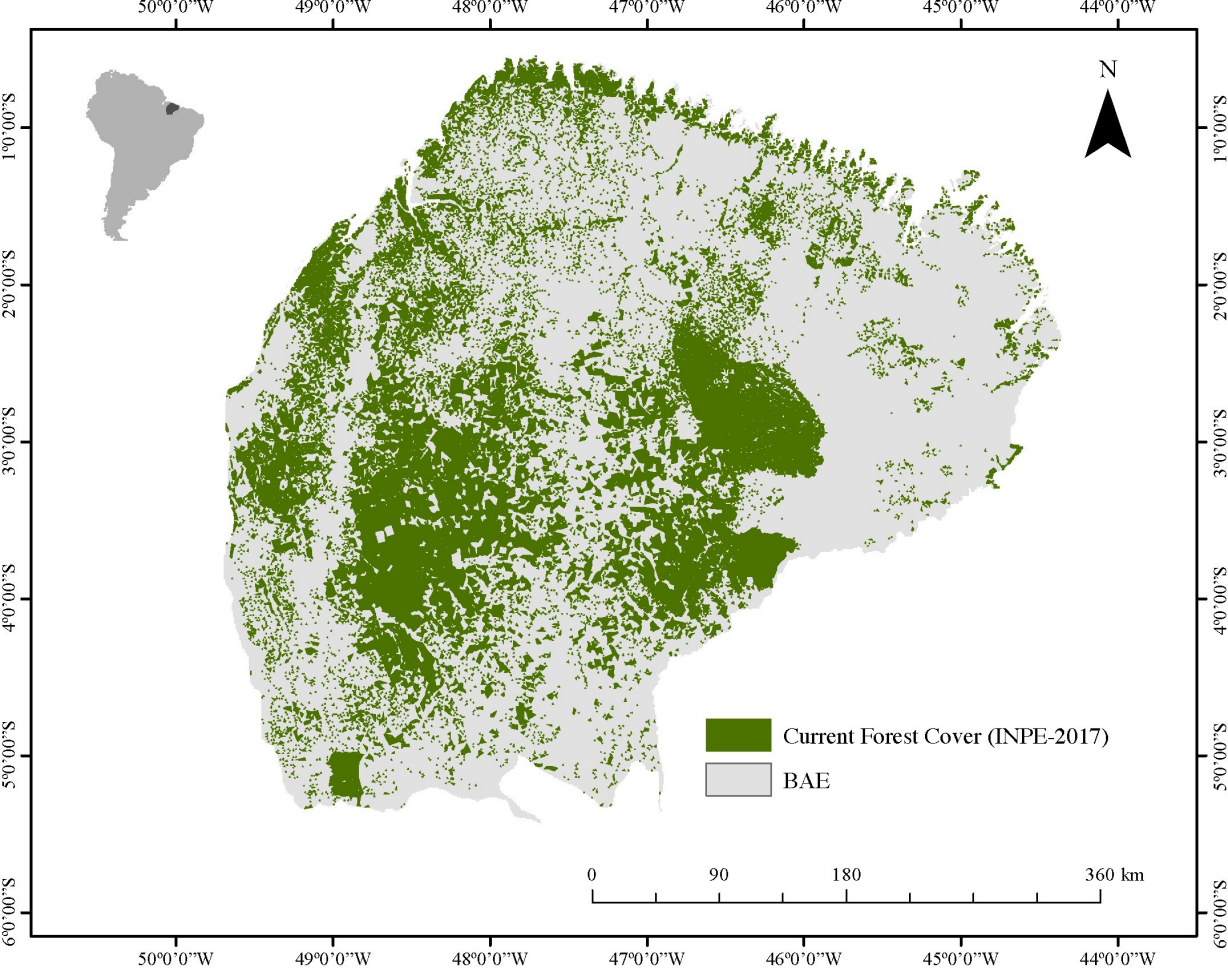
The Bélem area of endemism. Information on currently forested areas is from the National Institute for Space Research of Brazil (INPE).

The study was conducted in the Belém Area of Endemism (BAE), located in eastern Amazonia. BAE is the smallest of all Amazonian endemism centers with an extension of approximately 243,000 km^2^ that borders both eastern Pará (Tocantins River) and western Maranhão states (Pindaré River) [27] (Fig 1).

### Target taxa

As a selection criterion for the target taxa, we used all the 16 forest-dependent birds that inhabit the Belém area of endemism [55,56]. (Table 1).

**Table 1:** List of endemic species and subspecies occurring in the Belém area of endemism.

Taxa	English Name	ICMBio Threat Status [35]	IUCN Threat Status [34]
*Crax fasciolata pinima*	Belem Curassow	CR	CR
*Psophia obscura*	Black-winged Trumpeter	CR	CR
*Threnetes leucurus medianus*	Pale-tailed Barbthroat	-	LC
*Pteroglossus bitorquatus bitorquatus*	Eastern Red-necked Araçari	VU	EN
*Piculus paraensis*	Para Golden-green Woodpecker	EN	LC
*Thamnophilus aethiops incertus*	White-shouldered Antshrike	-	LC
*Phlegopsis nigromaculata paraensis*	Black-spotted Bare-eye	VU	LC
*Dendrocincla merula badia*	White-chinned Woodcreeper	VU	LC
*Dendrexetastes rufigula paraensis*	Cinnamon-throated Woodcreeper	EN	LC
*Synallaxis rutilans omissa*	Sooty Spinetail	-	LC
*Piprites chloris grisescens*	Wing-barred Piprites	VU	LC
*Terenotriccus erythrurus hellmayri*	Ruddy-tailed Flycatcher	-	LC
*Ramphocaenus melanurus austerus*	Long-billed Gnatwren	-	LC
*Granatellus pelzelni paraensis*	Rose-breasted Chat	-	LC
*Tangara velia signata*	Opal-rumped Tanager	VU	LC
*Lanio cristatus pallidigula*	Flame-Crested Tanager	-	LC

LC = Least Concern

VU = Vulnerable

EN = Endangered

CR = Critically Endangered

### Occurrence data

We compiled georeferenced occurrence records available at online databases such as GBIF (http://www.gbif.org/), eBird (https://ebird.org/home), Biodiversity Portal (https://portaldabiodiversidade.icmbio.gov.br/), SpeciesLink (http://splink.cria.org.br/) and Vertnet (http://www.vertnet.org/index.html). We also obtained distribution records from both primary and secondary literature (books, scientific articles, theses, and published reports), the ornithology collection of the Museu Paraense Emilio Goeldi and, previous work done by the Vertebrate ecology and zoology laboratory of the Federal University of Pará and fellow researchers. Occurrence data were evaluated by experts to remove misidentified taxa. Species nomenclature adopted herein follows the Brazilian Ornithological Registration Committee recommendation [57]. Then, we controlled for sampling bias on georeferenced data by removing duplicates records leaving a single record per pixel. To reduce autocorrelation in occurrence data and possible sampling bias, we used a thinning technique using the package spThin [58]. Afterward, we used the distance of a Moran’s I variogram that minimizes the spatial autocorrelation to define the thinning distance.

### Environmental variables

Data on the current climate scenario were obtained from the Worldclim platform (http://www.worldclim.org). We downloaded the 19 bioclimatic variables at a resolution of 30 seconds (~1km grid size) and performed a Pearson Correlation Test (pair-wise Pearson correlation test) to identify potential collinearity between these variables (S1 Table) using the *R software* v3.4.0 [59]. When the correlation between variables was high (r> | 0.8 |), we did not use one of them to generate the distribution model. Thus, we selected Average annual temperature (Bio1), Isothermality (Bio3), Temperature seasonality (Bio4), Annual precipitation (Bio12), and Precipitation of the wettest month (Bio13). To future climate scenarios (2050), we used the same variables selected for the current scenario and estimated models for 11 Atmospheric-Ocean General Circulation Models (BCC-CSM1-1, CCSM4, GISS-E2-R, HadGEM2-AO, HadGEM2-ES, IPSL-CM5A-LR, MIROC5, MRI-CGCM3, MIROC-ESM-CHEM, MIROC-ESM, NorESM1-M), which are all available at the Worldclim database. We considered both the optimistic (rcp45) and the pessimistic (rcp85) estimates of greenhouse gas emissions. The rcp45 depicts the mitigation scenario, while the rcp85 is the baseline *business-as-usual* scenario without additional efforts to restrain gas emissions [60].

### Modeling and model evaluation

Potential species distribution modeling was performed using three different algorithms to obtain more reliable predictions of the species distribution [61]. *Maximum Entropy* (MaxEnt) [62] is based on a presence-background approach that evaluates the relationship between the actual species occurrence and the entire study area [42]. On the other hand, Support Vector Machine (SVM) [63] and Random Forest (RDF) [64] algorithms are presence-absence models that use occurrence records and compare them with absence data. For algorithms that require pseudo-absences, we used the random space allocation method [65]. The number of pseudo-absences was equal to the number of presences for each species. In order to produce more accurate models, we use the bootstrap method [66] to partition data into 70% training and 30% testing sets and repeated this procedure five times. Species Distribution Modeling analysis included in the package ENMTML [67] scripts available on GitHub (github.com/andrefaa/ENMTML). All models were generated using the *R* software.

We evaluated models using the Sorensen index [68, 69], which ranges from 0 to 1. It is a threshold-dependent method where values closer to 1 indicate greater overall model accuracy; i.e., it has high precision and good performance [69]. Subsequently, we combined all model predictions for a species to obtain its final model (full ensemble) for both the present and future scenarios. We obtained the final ensemble through the weighted average of all generated models whose values were higher than the mean Sorensen values.

**Fig 2:**
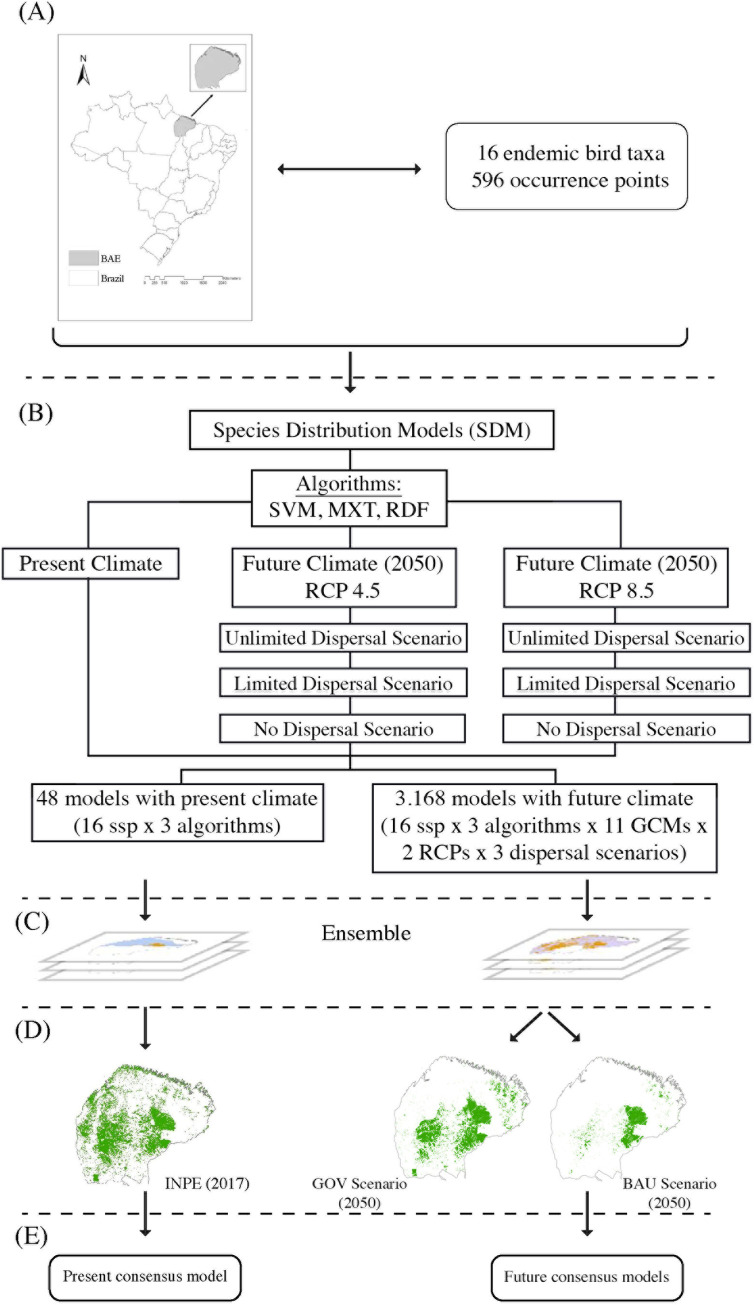
Concept map of the methods used in this work. (A) the study area and the taxa information used to construct the models. (B) The production of species distribution models from the RCPs, selection of climate variables, dispersal frameworks, and the algorithms used to produce the models. (C) the ensemble approach to generate a consensus model for both present and future scenarios. (D) the overlap of species distribution models with local current forest cover (2017) and two future deforestation scenarios (2050). (E) The endemic taxa final model for the Belém area of endemism (BAE).

Then, we applied a spatial restriction method based on species dispersal limitation [70, 71]. This approach—called here MSDM—relates the likelihood of a species’ occurrence at a given site to its distance from other localities where the species has been documented. In this method, we first created a layer of the summed distance from each cell to all occurrences that were later incorporated into the model fitting [70, 72]. This procedure is available in the script used in all of our modeling process (github.com/andrefaa/ENMTML) [67] (Fig 2).

### Overlap of species distribution models and deforestation models

Knowing that these are forest-dependent species, we overlapped the SDMs with forest remnants for both present and future scenarios (Fig 2). We obtained information on currently forested areas from the Brazilian Amazon Rainforest Satellite Monitoring Program (PRODES; http://www.obt.inpe.br/OBT/assuntos/programas/amazonia/prodes) from the National Institute for Space Research of Brazil (INPE). Data on future forest cover have been extracted from the SimAmazonia database (http://csr.ufmg.br/simamazonia/, [26]), which provides two types of deforestation scenarios: *business-as-usual* (BAU) and governance (GOV). The BAU scenario assumes that novel protected areas will not be created, a low compliance rate with current environmental laws, and a tendency for deforestation across the Amazon basin based on the historical deforestation rate and its variations from 1997 to 2002. On the other hand, GOV is an optimistic future deforestation scenario for 2050. It depicts a context where there would be more investments in sustainable development, greater compliance with environmental legislation, expansion of protected areas in the Amazon basin, besides taking satellite information into account in environmental licensing [26].

As our target taxa are dependent on forests, we expect that deforestation may affect the colonization potential of climatically suitable areas in the future since species tend to behave in different ways in the face of climate change and deforestation [24, 73]. Thus, we created different dispersal contexts to compare the effect of climate change on species distribution in the future, considering unlimited, limited, and no dispersal events. In the first framework, we consider unlimited dispersal, which allows species to colonize all new suitable environments. On the second one, dispersal is limited, thus reducing species ability to colonize the newly suitable areas. For this, we applied the MSDM method aforementioned. In the third scenario, we consider that the species cannot disperse into new suitable habitat, consequently being restricted to their current distribution. These three dispersal schemes combine either ‘optimistic’ or ‘pessimistic’ projections of climate change and deforestation. Therefore, our optimistic projections combine the three dispersal contexts with the rcp4.5 greenhouse gas emission model and the GOV deforestation model (Mitigation Scenario). In contrast, the pessimistic predictions combine them with the rcp8.5 greenhouse gas emission and the BAU deforestation models (*Business-as-usual* Scenario).

### Stacked species distribution models

To obtain species richness maps in the different environmental scenarios, we performed stacked-species distribution models (S-SDM) of all the species for both current and future projections. This method has already shown to be effective in several different situations [74, 75, 76]. For all stackings, we used the “raster calculator” tool available in QuantumGis 2.8 software.

## Results

We obtained a total of 596 unique occurrence records for the 16 target taxa with *Dendrexetastes rufigula paraensis* having the least number of occurrence records (7) and *Thamnophilus aethiops incertus* with the highest number of records (80) (S2 Table). The mean Sorensen index value was 0.71, ranging between 0.66 and 1 (S2 Table). The species *Threnetes leucurus medianus* was removed from the analysis because it presented results that may have been biased by the mathematical models in the dispersion calculation, due to the distribution of its occurrence records. By overlapping the taxon distribution models with the current and future forest remnant predictions for each climatic scenario, we observed an average loss of 73.80% of suitable areas in either in the mitigation and the *business-as-usual* scenarios (Table 2).

**Table 2:** Estimates of loss and gain of adequate areas in relation to climatic and deforestation impacts, considering the three dispersal frameworks. The mitigation scenario relates the optimistic climate projections (rcp45) to GOV’s predicted deforestation, whereas the *Business-as-usual* scenario relates the pessimistic climate (rcp85) to the BAU deforestation predictions. The numbers indicate the percentage of variation in the size of suitable areas in the future compared to currently suitable areas. Negative values (—) indicate loss while positive values (+) indicate gains of suitable areas.

Taxa	Mitigation Scenario			*Business-as-usual* Scenario		
	Unlimited Dispersal	Limited Dispersal	No Dispersal	Unlimited Dispersal	Limited Dispersal	No Dispersal
*Crax fasciolata pinima*	+59.57%	+59.57%	-73.54%	-99.89%	-37.08%	-80.24%
*Psophia obscura*	-9.45%	-97.19%	-99.74%	-88.22%	-99.26%	-99.97%
*Pteroglossus bitorquatus bitorquatus*	-13.59%	-13.59%	-73.20%	-100%	-42.25%	-79.74%
*Piculus paraensis*	+17.57%	-100.00%	-100.00%	-100%	-100.00%	-100.00%
*Thamnophilus aethiops incertus*	-100.00%	-82.07%	-85.61%	-89.49%	-77.26%	-81.83%
*Phlegopsis nigromaculata paraensis*	-26.46%	-28.02%	-58.71%	-92.96%	-69.67%	-93.84%
*Dendrocincla merula badia*	+14.95%	+14.95%	-65.66%	-99.98%	-53.96%	-87.56%
*Dendrexetastes rufigula paraensis*	-100.00%	-100.00%	-100.00%	-99.72%	-100.00%	-100.00%
*Synallaxis rutilans omissa*	-38.48%	-37.97%	-59.96%	-83.76%	-72.08%	-79.86%
*Piprites chloris grisescens*	-100.00%	-100.00%	-100.00%	-99.10%	-100.00%	-100.00%
*Terenotriccus erythrurus hellmayri*	-100.00%	-100.00%	-100.00%	-98.18%	-100.00%	-100.00%
*Ramphocaenus melanurus austerus*	-28.13%	-27.96%	-58.00%	-83.17%	-71.59%	-80.46%
*Granatellus pelzelni paraensis*	-99.85%	-94.07%	-99.44%	-100%	-94.13%	-94.13%
*Tangara velia signata*	+4.79%	+4.79%	-67.45%	-100%	-58.68%	-91.73%
*Lanio cristatus pallidigula*	-100.00%	-100.00%	-100.00%	-100%	-100.00%	-100.00%

Among the most threatened taxa according to the list of Brazilian endangered species [35], *Piculus paraensis* lost its total suitable area in five combinations of dispersal-climate-deforestation scenarios while *D*. *r*. *paraensis* was profoundly affected in all of the combinations (S1 Fig). Besides, *Psophia obscura*, which is considered “critically endangered,” according to IUCN [34], may also lose 68.67% and 95.82% of the adequate areas in the mitigation and the *business-as-usual* scenarios, respectively. *Lanio cristatus pallidigula* was the only taxon that we did not found future climatically suitable areas in any dispersal contexts or deforestation projections. Thus, this species faces a high risk of being extinct by the year 2050 (Table 2, S1 Fig).

We found variations between loss and gain of adequate areas for species in both the optimistic and pessimistic predictions. In the mitigation scenario, the average loss of suitable areas for the future is 59%. On the one hand, when analyzing the dispersal contexts in the mitigation framework (GOV), the unlimited dispersal scenario presents an attenuated impact on bird species compared to the other dispersal models, with an average loss of 41.27% of the adequate area. Surprisingly, when we assumed unlimited dispersal, four species have adequate area gains, with *Crax fasciolata pinima* (+59%) showing the most significant area increment. On the other hand, we found that average area loss was 12% higher when dispersal was limited, even though *C*. *f*. *pinima*, *P*. *paraensis*, *Dendrocincla merula badia*, and *Tangara velia signata* still showed the same percentages of area expansion in the unlimited dispersal scenario (Table 2, S1 Fig).

The no-dispersal framework shows the most extreme negative results in mitigation projections, where there was an average loss of 82% of suitable areas for the occurrence of the endemic birds, of which eight of them have losses above 80% of their current occurrence geographic range. We also did not found evidence of area expansion for any of the studied taxa (Table 2). Our results indicate that there is no adequate area for *D*. *R*. *paraensis*, *Piprites chloris grisescens*, and *Terenotriccus erythrurus hellmayri* in future mitigation scenarios.

The *business-as-usual* scenario shows that even considering three conditions of dispersal (Unlimited, Limited and No dispersal), there was a substantial reduction of adequate area (average: 88%) for all species, i.e., even in a scenario where species can freely disperse, there will be few suitable areas for the establishment of populations. In the *business-as-usual* deforestation projection (BAU) with unlimited dispersal, we found an average contraction of 95% of the suitable areas in which 11 species lost more than 90% of the adequate areas. Among these species that lost their entire suitable area are *Pteroglossus bitorquatus bitorquatus*, *P*. *paraensis*, *Granatellus pelzelni paraensis*, *Tangara velia signata*, and *Lanio cristatus pallidigula* (Table 2, S1 Fig). Moreover, these species also lost total habitat in the no-dispersal scenario, with an average loss of 90%. The limited dispersion context is the one that presents less drastic results compared to the other ones, since it indicates an average habitat loss of 78%, however, there was no suitable area expansion for any of the species.

**Fig 3:**
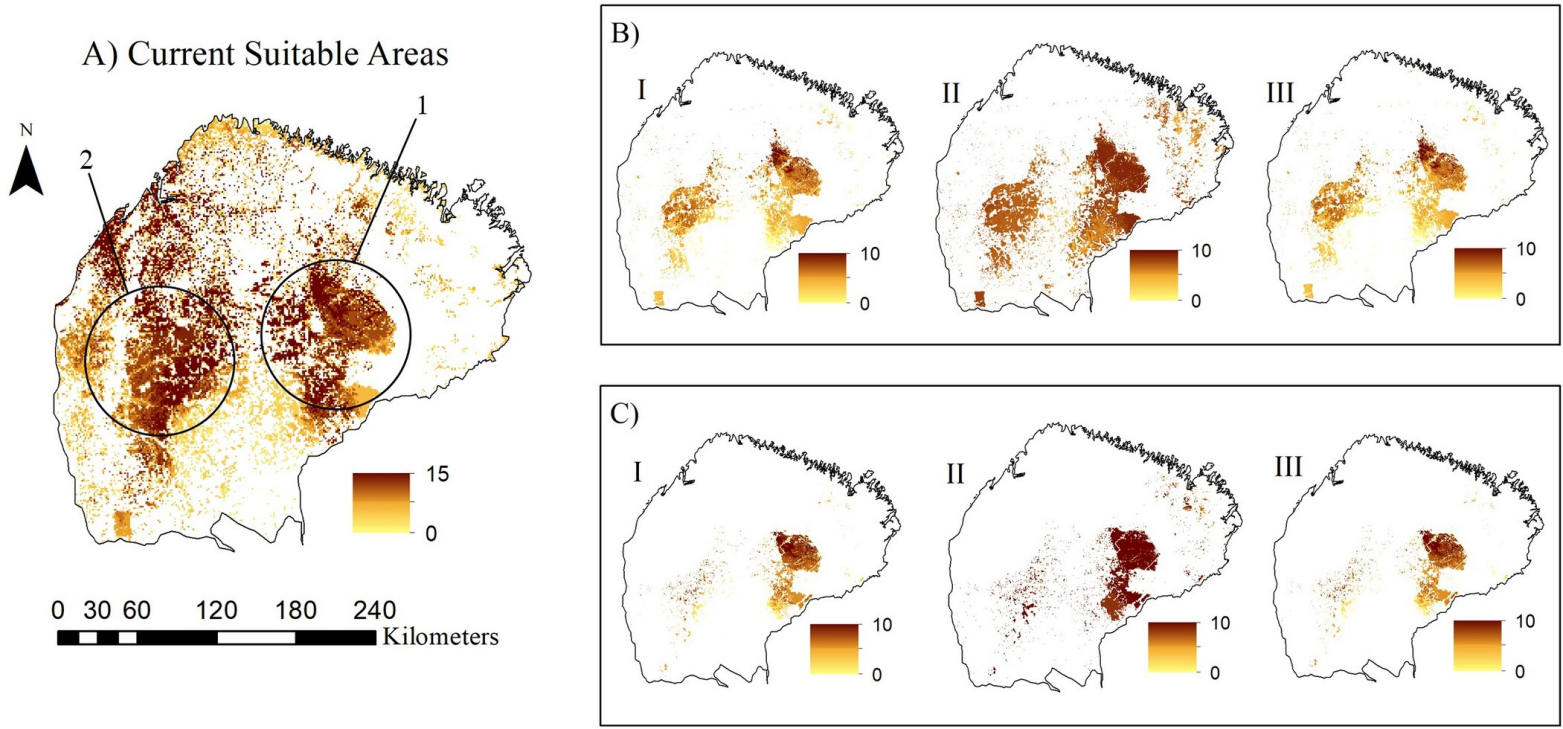
Maps of the potential distribution of endemic bird species from the Belém Area of Endemism. 1—“Gurupi Mosaic”; 2—“Rio-Capim” watershed. (A) represents the current climate suitability areas, while (B) represents the mitigation scenario, and (C) the *Business-as-usual* scenario. Regarding different dispersion contexts, sections (I), (II), and (III) demonstrate the Unlimited Dispersal, Limited Dispersal, and No Dispersal scenarios, respectively. Dispersal scenarios were applied to the projections. The mitigation and *business-as-usual* scenarios applied are projections constructed from perspectives of greenhouse gas emissions, and investments in conservation policies. Colors that vary between yellow and brown represent the number of species per cell in the projections. Both projections of forest remnants have been extracted from the SimAmazonia database [26].

The bird species richness per cell in BAE ranged from 0 to 15 and 0 to 10 in the current and future projections, respectively, due to the lack of environmental suitability in the dispersal frameworks we assumed herein. In both optimistic and pessimistic projections, species riches are mainly concentrated in the central-eastern region of BAE in all dispersal frameworks. In addition to the concentration of bird species in this region, the projections for the *business-as-usual* scenario indicate the existence of adequate area fragments in the southwest portion of the BAE (Fig 3). In contrast, in the mitigation scenario, species richness is concentrated mainly in the middle east, middle west, and south regions (Fig 3). Moreover, the results reveal a low concentration of suitable areas near the Tocantins River and BAE’s northern region in all scenarios.

The limited dispersal scenario indicates the existence of adequate area fragments in the northeastern region of the BAE in both mitigation and *business-as-usual* scenarios. Such circumstances suggest possible movements of species to this region since these areas harbor lower richness and distribution of species in the current climatic and deforestation conditions (Fig 3). The results also reinforce the importance of the protected areas existing in the BAE since projections predict the incidence of high species richness within or around these areas (Fig 3). Additionally, when comparing the currently appropriate areas for species to occur with the dispersal and deforestation scenarios, it is possible to note the occurrence of areas that can act as climatic refuges for the species, especially in the regions known as “Mosaico do Gurupi” and “Rio Capim” watershed (Area 1 and 2, Fig 3).

## Discussion

Our results indicate that BAE’s endemic birds will lose an average of 73% of their suitable areas by 2050. Moreover, at least six of these 15 bird species will have either less than 10% or even no suitable areas for their occurrence in all scenarios we analyzed. Since they are highly dependent on forest habitats, these species are already undergoing strong reductions in their habitat because of the high rates of deforestation in the BAE [27].

Based on the parameters to define the threat status of a species [77,78], the projections performed in this study indicate a probable change in the threat status of our target taxa. Among these parameters, the extent of occurrence and area of occupation of the taxa of the Belém endemism center may considerably decrease by 2050 (Table 2, S1 Fig). Habitat loss is another factor that determines the threat status of the species [77], and a reduction of adequate habitat implies reduced species distribution and enhances its risk of extinction [79]. This factor mainly affects species such as *C*. *f*. *pinima* and *P*. *obscura*, both currently classified as critically endangered (Table 1). Besides, our results support a general pattern of suitable area loss in both the mitigation and *business-as-usual* scenarios, which has also been found in several studies in recent years. The reduction of suitable habitat areas as a consequence of climate change has been reported not only for birds but also for a myriad of different organisms, such as mammals and plants [44,80,81]. Jetz et al. [82] evaluated the effect of climate change on birds worldwide and showed that the occurrence area of hundreds of species would reduce by 50%, and the most affected species would be those inhabiting in tropical regions. Anciães & Peterson [83], who studied the effect of climate change on 49 species of manakins (Pipridae), found that at least 20% of all species will be locally extinct from their current distribution, and species occurring in the Amazonian lowlands would be the most affected. Thus, the species of birds that would be most affected by climate change tend to be those that occur in tropical regions, especially those that live in lowlands and have restricted spatial distribution, such as the birds that occur in BAE.

Although climatic fluctuations in the tropical region are smaller compared to other regions [84], the high level of Amazonian species specialization may accelerate the consequences caused by changes in climate [85], which reduces their chances to adapt to such novel environmental circumstances [38,82,86]. Additionally, deforestation is determinant in habitat loss [87], and becomes an aggravating factor for BAE’s taxa, as they are highly dependent on forest environments. These factors combined with anthropogenic activities, restricted distributions, and low dispersal ability, in virtue of species endemicity, can lead to severe population declines and, consequently, the loss of genetic variability [88], exposing the taxa to a higher risk of extinction. Assuming that a loss of suitable areas over 95% means a high risk of extinction [83], then at least 40% of our target species would be extinct by 2050 in any dispersion scenario (Table 2).

The predicted loss of suitable areas for BAE’s endemic taxa can culminate in considerable losses of ecological services—e.g., seed dispersal—and maintaining the forest itself [89,90]. For instance, frugivores species such as *P*. *b*. *bitorquatus*, *C*. *f*. *pinima* e *T*. *v*. *signata*, which are endemic to BAE [91, 92, 93] and play a key role on dispersing seeds from this region, is predicted to lose 44,57% of suitable habitat areas in future scenarios (Table 2, S1 Fig). Moreover, climate may directly influence the metabolism and reproductive success of birds [94,95] because it influences the incubation rates since high temperatures can reduce reproductive success [96]. Also, forest birds are particularly vulnerable to landscape changes; thus, many species are now restricted to smaller areas as a result of deforestation, and this has broader implications for ecosystem function [89].

The association between climate change and reduced habitat suitability has been a determinant factor in population reductions of various species worldwide, especially when it comes to endemic or species that have restricted distribution [82,97]. One of the main mechanisms used to mitigate the impacts of climate change on these species in the near future is to assess the current system of protected areas. It is necessary to ensure that these areas will continue being effective in conserving these species even under climate change [98,99,100]. Thus, the challenge is to assess whether protected areas that are already established will be appropriate for the bird species in the future, or whether we will have suitable forest remnant patches in the areas that are not protected. Despite the existence of protected areas in the studied region, their effectiveness in conserving bird species is low. Only 20% of the taxon distribution falls within the protected areas in the Belém Area of Endemism. Such low effectiveness may be related to the low percentage of protected areas existing within the Belem Area of Endemism [30]. Approximately 3,100 km^2^ of protected forests were cleared in 2016, a loss of 17.2% of their original extension [101], therefore further impacting species occurrence within the protected lands in the region. Such high levels of deforestation can affect mainly taxa in the future scenario since, in all projections (mitigation and *business-as-usual*) and multiple dispersal scenarios, the highest concentration of co-occurring species are within or around the protected forests (Fig 3).

The “Gurupi Mosaic” (Area 1 of Fig 3), which includes the Gurupi Biological Reserve (only federal UC of Integral Protection in BAE) and a complex of six Indigenous lands (Alto Turiaçu, Awá, Caru, Arariboia, Pindaré River and Alto Guamá River) stands out for the protection of BAE’s endemic bird species in all the dispersal frameworks in both mitigation and *business-as-usual* projections. This mosaic houses the largest remnant of the Amazon forest in BAE; thus, it is of paramount biological and socio-environmental importance. Also, inside this area, there is a high diversity of plants and animals, which includes more than 46 endemic and endangered species [102]. The taxa assessed in the present work are found within this complex, including the critically endangered *C*. *pinima* and *P*. *obscura* [103,104] Additionally, *Chiropotes satanas* and *Cebus kaapori*—the latter being among the 25 species that are at the highest risk of extinction of the World—can also be found in this region [105,106]. Despite its importance for conservation, the “Gurupi Mosaic” has undergone a dramatic reduction in forest cover due to the significant increase in illegal logging [103].

The “Rio-Capim” watershed is also an area that also deserves some attention. This region is a complex formed by private properties that also protect a large portion of the forest remnants of the BAE (Area 2 of Fig 3). Currently, Brazil has 53% of its forest areas in private properties [107], which increases the importance of these areas for the maintenance of forest-dependent fauna [108,109]. These areas can play an important role, functioning as stepping stones [110] and ecological corridors for all endemic species, as they favor their movement across the landscape, therefore functioning as complementary conservation areas [111,112]. These ecological corridors are relevant for conservation, to connect populations inhabiting fragmented regions [111,113], facilitate gene flow between populations, and ensure species dispersal [88].

These two large areas, identified through our analyses as climate refuges, are a good alternative for the conservation of BAE’s species, as they are areas of climatic stability, housing species and allowing their persistence in case of niche changes [51,114]. The climatic refuges of the BAE are mainly concentrated within protected areas, which reinforces their importance as main regions for the conservation of species [115,116,117]. However, few areas could serve as ecological corridors between these two areas. Thus, conservation efforts should be directed to the maintenance and preservation of these two significant remnants of vegetation in addition to the establishment of ecological corridors between them.

## Supporting information

S1 Fig Suitability models for endemic birds of the Belem Area of Endemism (BAE) on both climate-deforestation scenarios. Black lines and white area delimits the BAE region. Areas in yellow represents the potential distribution of each taxon based on current and future scenarios. From left to right, maps indicate, respectively: Potential distribution for the present-time, potential distribution in a mitigation scenario with unlimited dispersal (Mit_Unlim_Disp), potential distribution in a mitigation scenario with limited dispersal (Mit_Lim_Disp), potential distribution in a mitigation scenario with no-dispersal (Mit_No_Disp), potential distribution in a *Business-as-usual* scenario with unlimited dispersal (BAU_Unlim_Disp), potential distribution in *Business-as-usual* scenario with limited dispersal (BAU_Lim_Disp), potential distribution in a *Business-as-usual* scenario with no-dispersal (BAU_No_Disp).
(PDF)

S1 Table Worldclim bioclimatic variables table. (XLSX)

S2 Table Species distribution models data information. (XLSX)
