# Correction: Climate change and bird extinctions in the Amazon

**DOI:** 10.1371/journal.pone.0252260

**Published:** 2021-05-20

**Authors:** Kauê Felippe de Moraes, Marcos Pérsio Dantas Santos, Gabriela Silva Ribeiro Gonçalves, Geovana Linhares de Oliveira, Leticia Braga Gomes, Marcela Guimarães Moreira Lima

The following information is missing from the Funding statement: The publication of this article was supported by the Federal University of Pará (UFPA) (PROPESP-PAPQ 01/2020—QUALIFIED PUBLICATION SUPPORT PROGRAM).

## References

[1] de Moraes KF, Santos MPD, Gonçalves GSR, de Oliveira GL, Gomes LB, Lima MGM (2020) Climate change and bird extinctions in the Amazon. PLoS ONE 15(7): e0236103. 10.1371/journal.pone.0236103 32678834 PMC7367466

