# Supplementary figures and images for: Climate change and bird extinctions in the Amazon
Source: PLoS One. 2020 Jul 17;15(7):e0236103. doi: 10.1371/journal.pone.0236103 (PMC7367466; doi:10.1371/journal.pone.0236103)

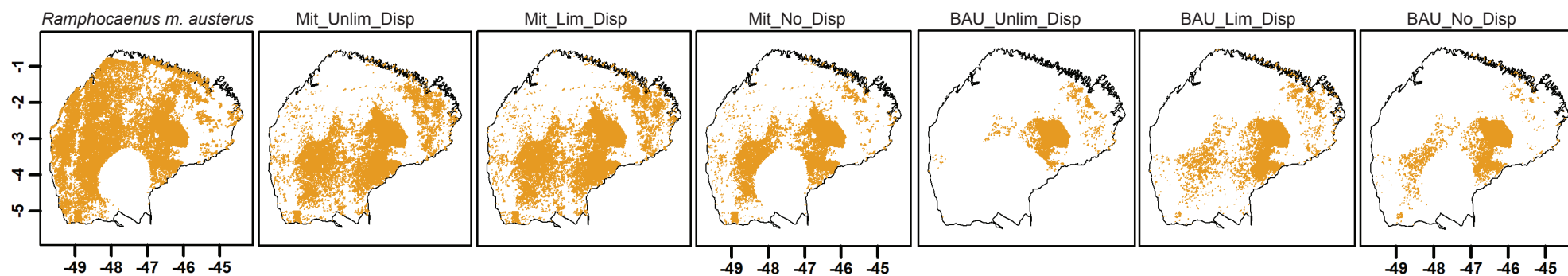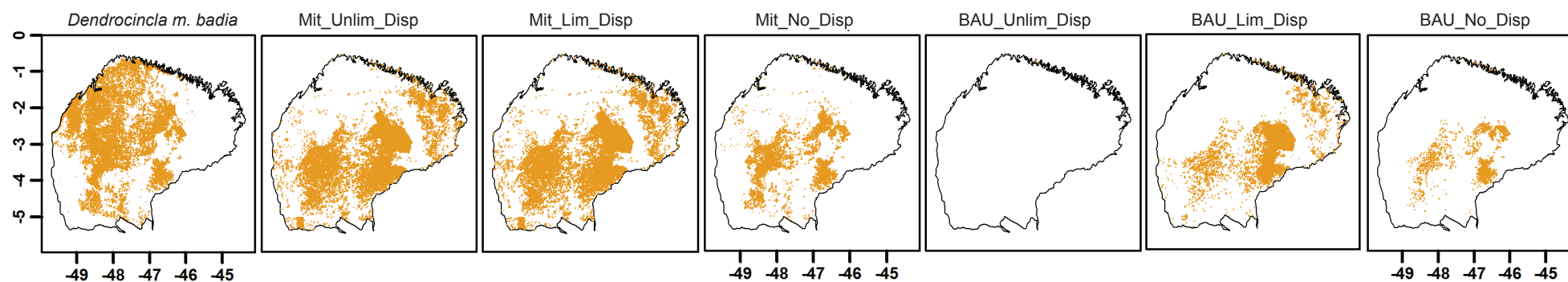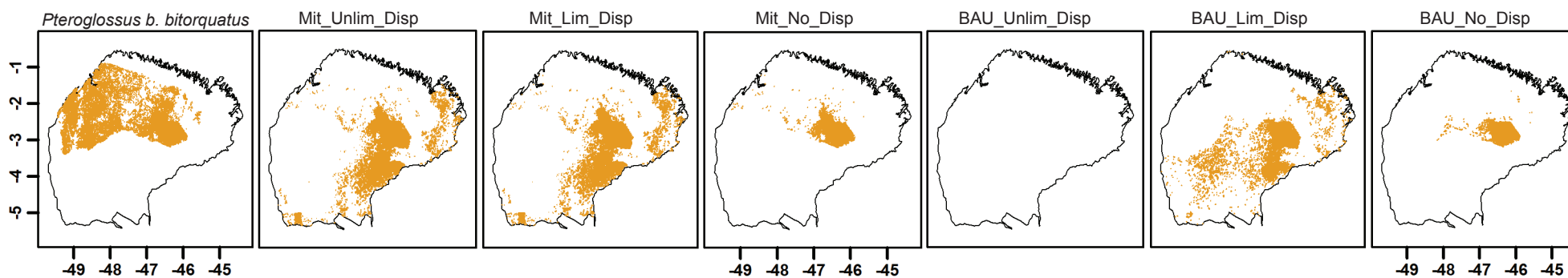

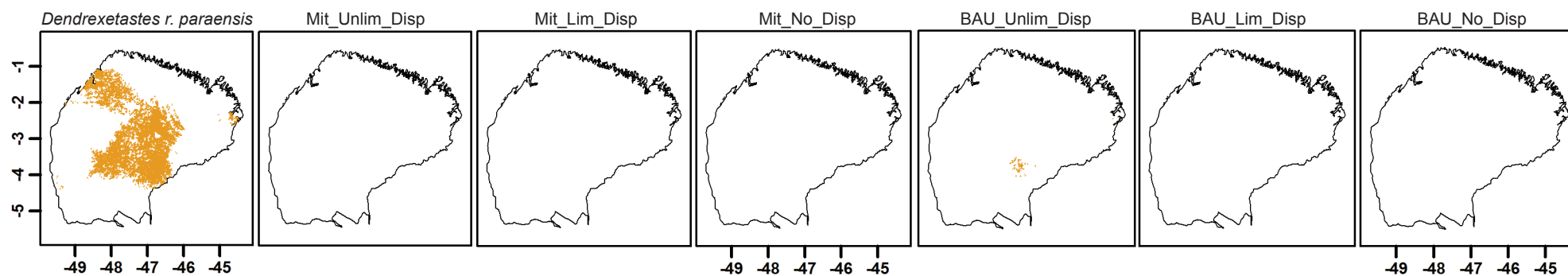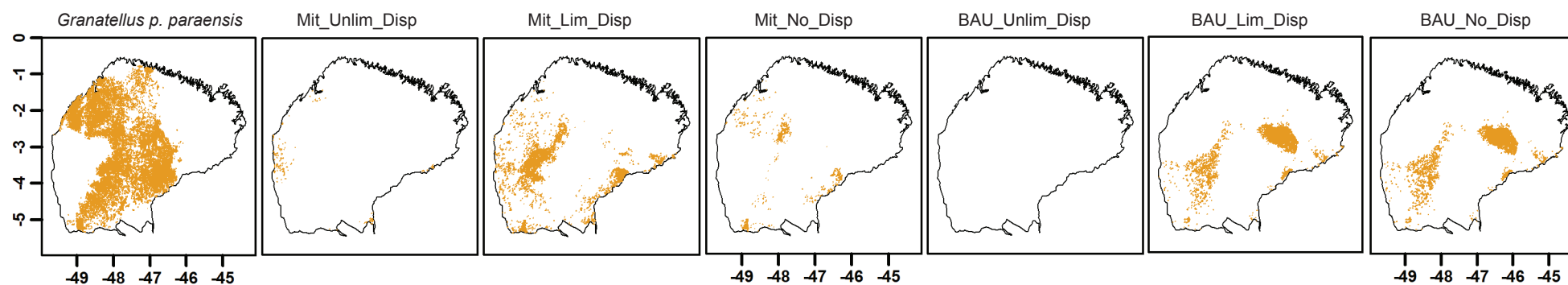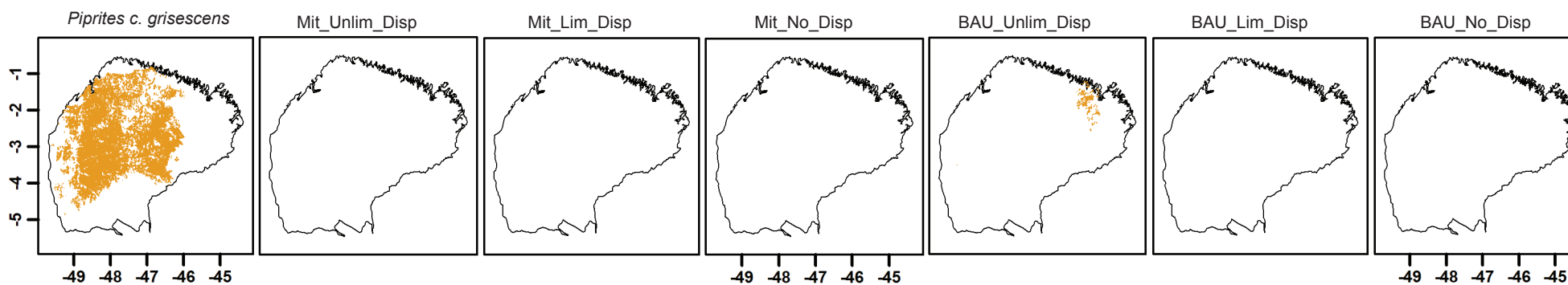

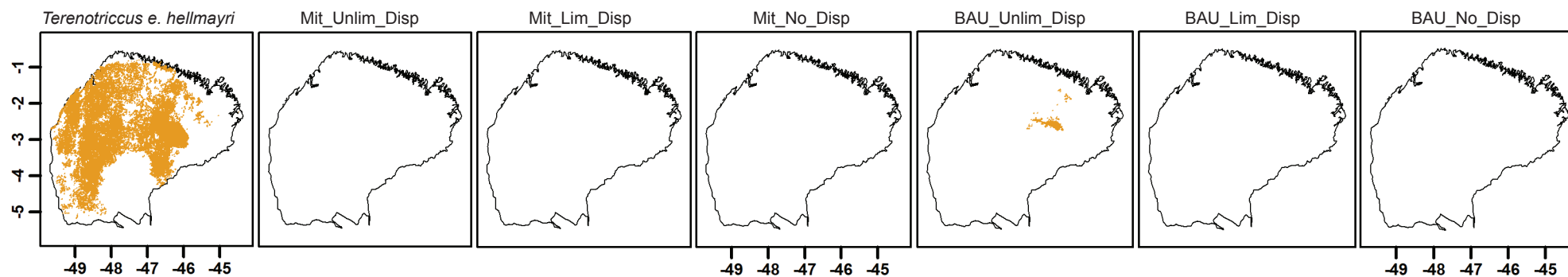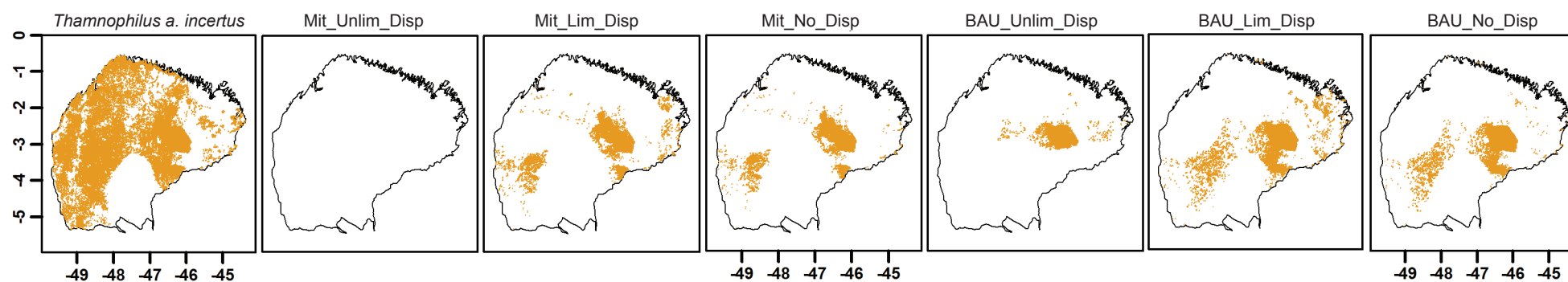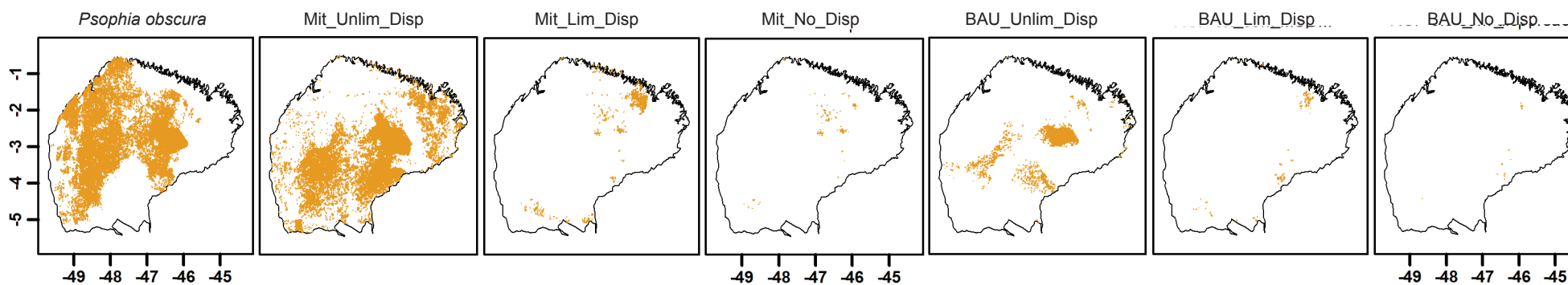

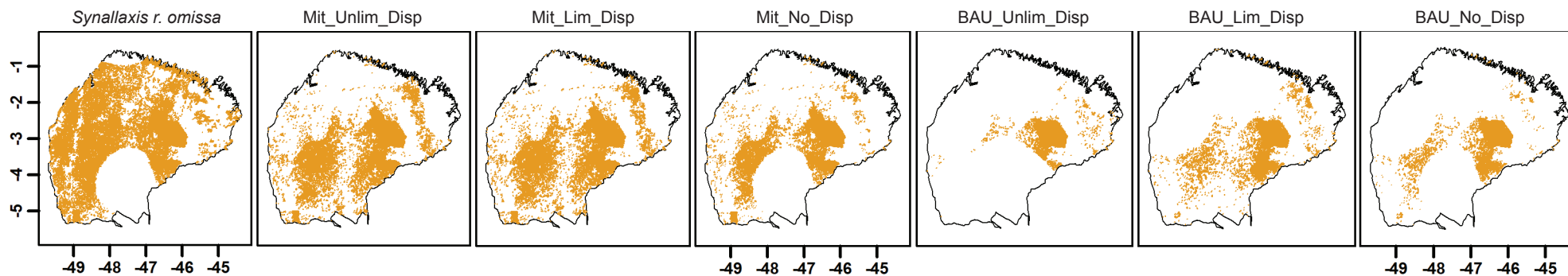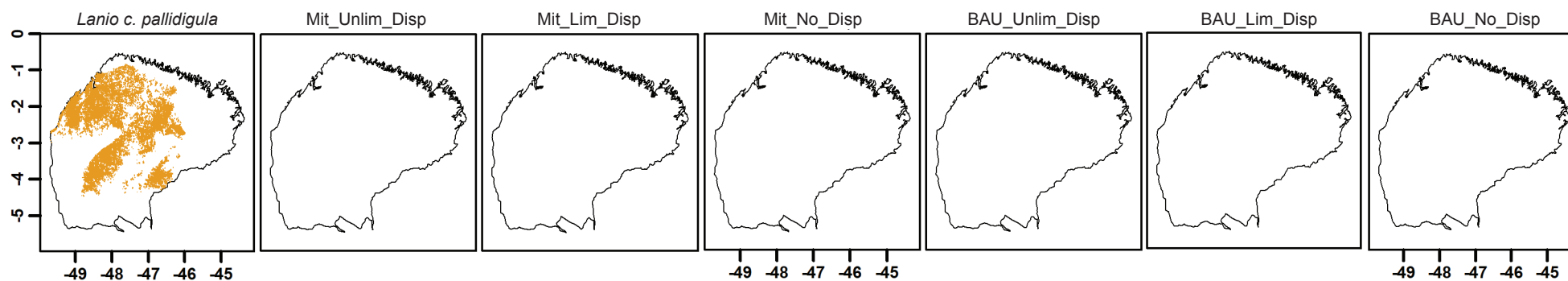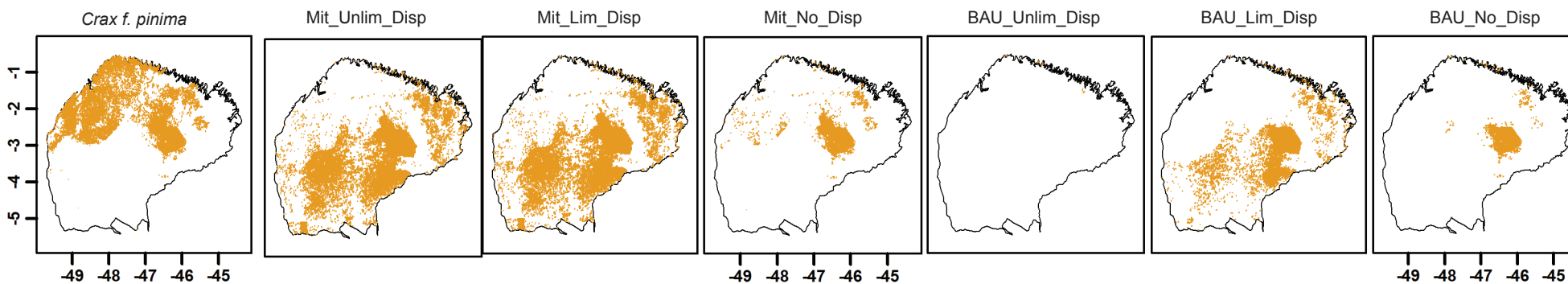

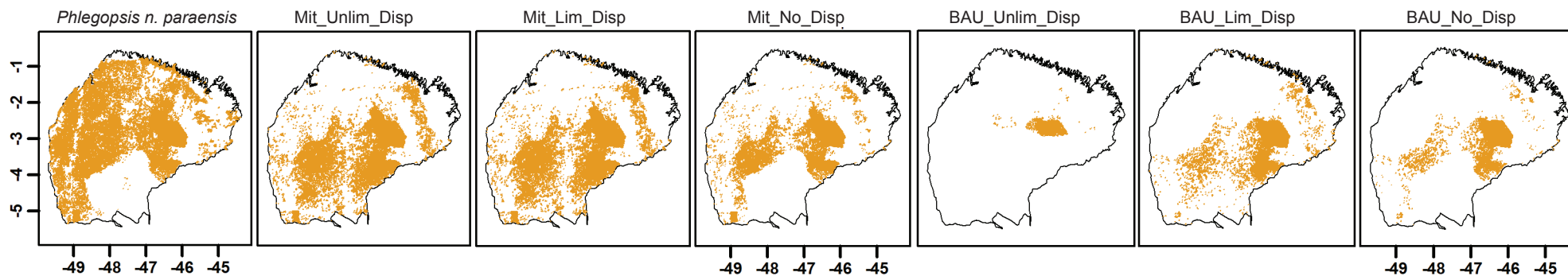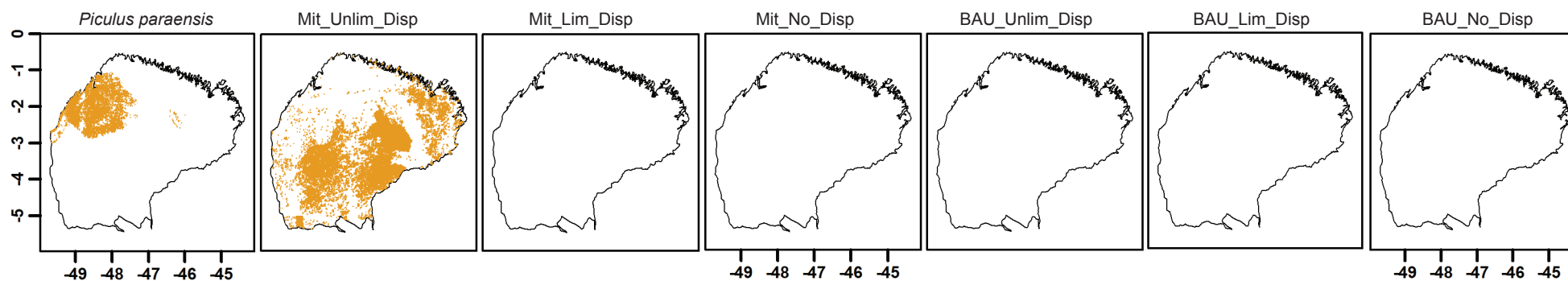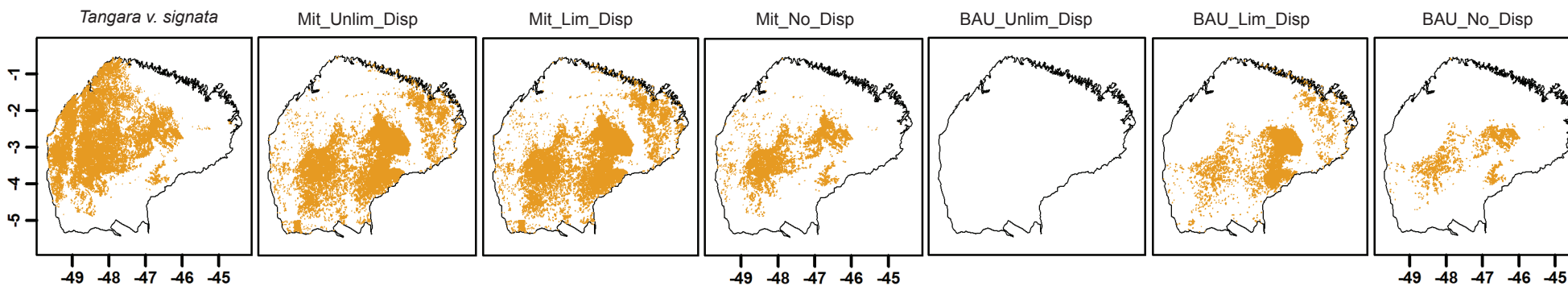

Supplement: S1 Fig — Black lines and white area delimits the BAE region. Areas in yellow represents the potential distribution of each taxon based on current and future scenarios. From left to right, maps indicate, respectively: Potential distribution for the present-time, potential distribution in a mitigation scenario with unlimited dispersal (Mit_Unlim_Disp), potential distribution in a mitigation scenario with limited dispersal (Mit_Lim_Disp), potential distribution in a mitigation scenario with no-dispersal (Mit_No_Disp), potential distribution in a Business-as-usual scenario with unlimited dispersal (BAU_Unlim_Disp), potential distribution in Business-as-usual scenario with limited dispersal (BAU_Lim_Disp), potential distribution in a Business-as-usual scenario with no-dispersal (BAU_No_Disp). (PDF) [file pone.0236103.s001.pdf]
